# Transcription Factor-Based Genetic Engineering in Microalgae

**DOI:** 10.3390/plants10081602

**Published:** 2021-08-04

**Authors:** Keiichi Mochdia, Shun Tamaki

**Affiliations:** 1 RIKEN Center for Sustainable Resource Science, Tsurumi-ku, Yokohama 230-0045, Japan; 2 Kihara Institute for Biological Research, Yokohama City University, Totsuka-ku, Yokohama 244-0813, Japan; 3 RIKEN Baton Zone Program, Tsurumi-ku, Yokohama 230-0045, Japan; shun.tamaki@riken.jp; 4 School of Information and Data Sciences, Nagasaki University, Bunkyo-machi, Nagasaki 852-8521, Japan

**Keywords:** whole genome assembly, transcription factors, gene regulatory networks, microalgae

## Abstract

Sequence-specific DNA-binding transcription factors (TFs) are key components of gene regulatory networks. Advances in high-throughput sequencing have facilitated the rapid acquisition of whole genome assembly and TF repertoires in microalgal species. In this review, we summarize recent advances in gene discovery and functional analyses, especially for transcription factors in microalgal species. Specifically, we provide examples of the genome-scale identification of transcription factors in genome-sequenced microalgal species and showcase their application in the discovery of regulators involved in various cellular functions. Herein, we highlight TF-based genetic engineering as a promising framework for designing microalgal strains for microalgal-based bioproduction.

## 1. Introduction

Sequence-specific DNA-binding transcription factors (TFs) are key components of gene regulatory networks [1,2,3,4,5]. By binding to specific DNA sequence motifs, TFs function as molecular switches that regulate the transcription of multiple target genes, resulting in phenotypic changes [6]. TFs form complex regulatory networks through specific interactions with cis-regulatory sequences of promoter regions of target genes, as well as interactions with other proteins, including transcriptional regulators, such as chromatin remodeling/modifying factors [7,8]. Such specific TF-promoter interactions mediate molecular signals and regulate gene expression, which affects cellular metabolism, homeostasis, and physiological responses to environmental changes.

In plants, TFs are distributed from unicellular aquatic algae to complex land plants [9]. Functional analyses of genes encoding TF proteins in model plants, such as *Arabidopsis*, revealed their critical regulatory functions involved in gene regulatory networks underpinning their growth, development, and environmental responses [10,11,12]. Through molecular genetics, mutations in TF-encoding genes have been identified in crops that are often involved in the domestication process, accompanied by critical phenotypic changes in agronomic traits [13,14]. Moreover, recent remarkable advances in whole-genome sequencing have enabled us to explore the full set of TF–encoding genes and to further narrow down key regulators in the gene regulatory network in various organisms. Information resources for TFs, comprehensively identified from various plant genomes based on family assignment rules for plant TFs, have also facilitated the elucidation of TF functions [15,16,17,18,19]. Moreover, recent advances in high-throughput sequencing technologies have led to the development of various applications, such as ATAC-seq (assay for transposase-accessible chromatin with high-throughput sequencing) and TF Chip-seq, to analyze epigenomic landscapes that have enabled the assessment of TF–promoter interactions [20,21].

By increasing the number of genome-sequenced microalgal species, TFs comprehensively identified from algal genomes offer opportunities to explore key regulators involved in various cellular functions in microalgae, such as cellular metabolism and environmental responses. In this review, we present the current status of the genome-scale identification of TF-encoding genes in microalgae and showcase recent examples of engineering gene regulatory networks in microalgal species. Subsequently, we discuss perspectives in gene regulatory network (GRN) engineering to improve the productivity and adaptation ability of microalgae to environmental changes.

## 2. Whole Genome Assembly in Microalgae

Whole-genome assembly data are an indispensable information resource for basic and applied research, even with respect to microalgal species. Data from gene models annotated in a whole-genome assembly provide a framework that facilitates genome-scale studies, including genome-wide identification of gene families, such as TF repertories in a particular genome-sequenced model plant species, such as *Arabidopsis thaliana* [22]. In the early 2000s, genome sequencing efforts also deciphered the entire genetic codes of some eukaryotic microalgae, such as *Cyanidioschyzon merolae* [23], the diatom *Thalassiosira pseudonana* [24], and *Chlamydomonas reinhardtii* [25], through the assembly of Sanger-sequencing-based reads of plasmid or BAC/fosmid-end sequencing and gene prediction with EST and full-length cDNA evidence. Since next-generation sequencers were commercially launched around 2005 (so-called second-generation sequencers), massive parallel sequencing methods have expedited the construction of whole-genome assemblies in various organisms, including microalgal species. The combinatorial use of sequence data from multiple sequencing methods facilitates de novo whole-genome assembly in multiple microalgae. For example, the *Cyanophora paradoxa* genome assembly was constructed using sequencing data through multiple sequencing methods, such as pyrosequencing, sequence-by-synthesis, and Sanger sequencing [26]. For the de novo assembly of smaller genomes, such as the genome of the red alga *Porphyridium purpureum*, an Illumina-based sequencing method was applied to determine its ca. 20-Mbp genome [27]. Single-molecule DNA sequencing technologies (so-called third-generation sequencers) have emerged over the past decade, and have provided technologies for long-read sequencing (e.g., >200 kb in the maximum read length in the continuous long-read mode of Pacific Biosciences (PacBio) Sequel II, and >1500 kb in the ultra-long mode of Oxford Nanopore Technologies (ONT) [28]), enabling us to assemble highly contiguous genome sequences. A hybrid assembly strategy, using PacBio-based long reads and Illumina-based short reads for the error collection of the PacBio reads, was applied to decipher the genome sequences of *Chlorella sorokiniana* UTEX 1602 and *Micractinium conductrix* SAG 241.80, and to compare their genomic components [29]. Moreover, recently developed physical mapping technologies, such as chromatin conformation capture (Hi-C) and optical mapping, have also provided promising approaches to improving contiguity in the sequence assembly, which enables chromosome-scale assembly [30,31].

Over the past two decades, a number of sequencing projects have deciphered and annotated various algal genomes. There have been some excellent studies that have reviewed published genome-sequenced algae [22,32]. According to PhycoCosm (https://phycocosm.jgi.doe.gov, accessed on 16 June 2021), an algal genomic information portal provides more than 100 algal genomes and annotations [33]. A recent review by Hanschen and Starkenburg assessed the current status of algal genome resources and identified more than 200 genome assemblies by reviewing multiple information resources for algal genomes [34]. In their review, Hanschen and Starkenburg highlighted a trend of the declining quality of genomic resources, represented by a reduction in the assembly quality, gene annotation quality, and genome completeness, and suggested that the combinatorial use of multiple sequencing and mapping technologies may improve their quality. Using a hybrid approach with Illumina, PacBio, and optical mapping (OpGen), Roth et al. succeeded in yielding a high-quality pseudochromosome assembly of the *Chromochloris zofingiensis* genome (~58 Mbp, 19 chromosomes) [35]. Using another hybrid combination of PacBio, Illumina, and Hi-C, the whole-genome assembly of *Nannochloropsis oceanica*, which was composed of 32 pseudochromosomes, suggested that most of its annotated genes may originate from the nucleus of a red alga symbiont through secondary endosymbiosis [36]. The combined use of multiple sequencing technologies would allow us to improve previously published assemblies, as well as to construct chromosome-scale assemblies in new algal species and strains.

## 3. Genome-Wide Identification of TF Repertories in Microalgae

Since the first genome-scale identification of TF-encoding genes in *Arabidopsis* was published [22], the whole-genome assemblies have allowed us to identify TF-encoding genes based on the conserved sequence profiles of DNA-binding domains and to develop information resources that provide the detailed annotation of TFs in various plant species [15,16,17,18,19]. Such information resources regarding TFs (the so-called TF database) have facilitated the identification of transcriptional regulators and the elucidation of their regulatory roles in various functions, such as growth and development [12], their environmental stress responses [2,11], disease resistance [3,37] in model plant species, and various agronomic traits in crop species [17,38,39]. Moreover, the set of full-length cDNAs encoding TFs has also been useful for developing bio-resources in relation to experimental mutant lines with modified TF functions for the functional characterization of TFs [40,41,42] and to identify their interactors through high-throughput screening, such as yeast one-/two-hybrid screening for the elucidation of transcriptional regulatory networks in plants [43,44].

The family assignment rules for TFs have also been used to identify putative TF-encoding genes, even in microalgae. A study of the genome-scale identification of TF-encoding genes identified 234 genes encoding 147 TFs and 87 TRs of ~40 families in *Chlamydomonas* [45]. In that study, the authors used information on TFs previously identified in representative plant species, as well as other model organisms, to identify TFs and transcriptional regulators common to all eukaryotes. Thiriet-Rupert et al. applied a strategy that combined similarity search and DBD(DNA binding domain) search to identify TFs from seven algal genomes, and illustrated the specific properties of TF repertories across various algal lineages [46]. PlantTFDB (http://planttfdb.gao-lab.org/, accessed on 16 June 2021), a comprehensive information resource for TF families in green plants, provides TFs in 16 Chlorophyta and one Charophyta genome, identified based on a conserved domain-based family assignment rule [19]. PhycoCosm (https://phycocosm.jgi.doe.gov, accessed on 16 June 2021), a portal information resource for algal research, integrates genomic information in more than 100 algal species, and provides comparative profiles in gene families, including TF families, based on Pfam models associated with DBDs [33]. Since stable transgene expression, miRNA-based gene silencing, and targeted DNA editing are available in *Chlamydomonas*, mutant resources focusing on TF-encoding genes may provide useful tools for the functional characterization of TFs and their transcriptional regulatory networks in *Chlamydomonas*.

## 4. Gene Regulatory Networks with TFs

TF-encoding genes are often assumed to be regulators that affect the expression levels of other genes linked to the TF-encoding genes in computationally inferred gene-regulatory networks. Over the past two decades, many transcriptional networks have been constructed based on the correlated expression of genes in various species by analyzing a large number of microarray-based and RNA-seq-based transcriptome datasets (so-called gene co-expression networks) [47]. Although gene co-expression networks do not suggest regulatory causalities between genes, TF-encoding genes in a co-expression network imply that TFs might regulate other co-expressed genes, which facilitates the narrowing-down of candidate regulators involved in particular gene regulatory networks for further analyses. Such a gene co-expression-network-based approach succeeded in identifying key regulators involved in various plant functions, such as metabolism [48] and development [49]. Increasing the number of publicly available transcriptome datasets in model plants and even in crops, there are information resources that represent gene co-expression networks in plant species, such as ATTED-II (https://atted.jp/, accessed on 16 June 2021) [50], ALCOdb (http://alcodb.jp/, accessed on 16 June 2021) [51], and PlaNet (http://aranet.mpimp-golm.mpg.de/, accessed on 16 June 2021) [52]. ChlamyNET (http://viridiplantae.ibvf.csic.es/ChlamyNet/tutorial.html, accessed on 16 June 2021) provides a web-based tool to explore the co-expression networks in *Chlamydomonas*, based on 50 RNA-seq samples of eight genotypes under various physiological conditions [53]. ChlamyNET provides a user interface to search for gene families using PFAM domain identifiers, as well as gene set enrichment analyses regarding gene ontology (GO) terms and TF binding site motifs, which facilitate insights into the regulatory functions of TFs in the *Chlamydomonas* transcriptome. To date, over 1800 RNA-seq-based transcriptome datasets (Illumina RNA-seq datasets, available in NCBI SRA, accessed on 21 June 2021) are publicly available in relation to *Chlamydomonas*, which represents its transcriptome in response to various conditions. More recently, over 500 RNA-seq samples from 58 experimental series were generated to identify the gene co-expression network in *Chlamydomonas* [54]. Moreover, recently, through the 10X Genomics chromium-based library preparation of batch-cultured *Chlamydomonas* cells, single-cell RNA-seq analyses illustrated the cell types of *Chlamydomonas* cells under various conditions, including the diurnal cycle [55]. In addition to such co-expression-network-based approaches, statistical and machine learning-based gene regulatory network inference may provide a promising approach to infer causalities between TFs and their potential target genes, using the large-scale collection of transcriptome datasets from time-series and/or single cell samples [56]. By combining transcriptome data with molecular genetics tools and mutant resources [57,58], gene discovery and functional analysis will be further accelerated in *Chlamydomonas*, which will advance our understanding of transcriptional regulatory mechanisms underpinning its cellular functions, such as metabolism and environmental responses.

## 5. TF-based Metabolic Engineering in Microalgae

The manipulation of the cellular metabolism of microalgae is a long-standing challenge in microalgal biotechnology for the engineering of photobioreactors, in order to produce commodities and high-value chemicals. The combinatorial use of transcriptome, proteome, and/or metabolome analyses, and multi-omics-based approaches has presented some candidate regulators that mediate the transcriptional remodeling involved in lipid metabolism in *Chlamydomonas* [59,60]. For example, Jia et al. demonstrated that the overexpression of a gene encoding a DofF transcription factor in *Chlamydomonas* significantly enhanced its intracellular lipid content [61]. Some TF-encoding genes have been identified as regulators involved in stress responses, accompanying lipid-remodeling, which is one of the important strategies in plants used to adapt to environmental changes [62]. Bajhaiya et al. demonstrated that the Pi starvation response 1 (PSR1), a Myb transcription factor, regulate phosphorus starvation-induced lipid and starch accumulation in *Chlamydomonas*, suggesting its transcriptional regulation in global metabolism [63]. More recently, through a gene co-expression analysis of the transcriptome under phosphorus (P)-depleted conditions, Hidayati et al. identified that lipid remodeling regulator 1 (LRL1), homologous to AtMYB64, may regulate cellular responses, with lipid remodeling in response to P-depleted conditions in *Chlamydomonas* [64].

As another example, Yamaoka et al. identified a bZIP transcription factor involved in the ER stress response through lipid remodeling in *Chlamydomonas* [65].

Transcription factor-based genetic engineering has also considerably advanced in the engineering of lipid metabolism in other microalgal species. In *Nannochloropsis gaditana*, Ajjawi et al. identified a transcription factor that was homologous to Zn(II)2Cys6-encoding genes from 20 transcription factors that may negatively regulate lipid production, and demonstrated that, compared to the wild type, its knockout mutants produced twice as much of the lipid [66]. In *Nannochloropsis oceanica*, Li et al. identified a bZIP1 transcription factor, NobZIP1, and demonstrated that its overexpression enhanced lipid accumulation and secretion. These examples suggest that such transcription factor-based genetic engineering approaches are a promising strategy in the discovery of genes that are useful for manipulating cellular metabolism in microalgae.

## 6. Conclusions and Future Perspectives

**Figure 1:**
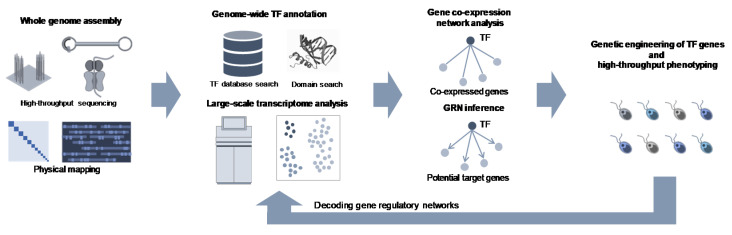
A schematic representation of transcription factor-based metabolic engineering in microalga species.

In this review, we have summarized the recent advances in gene discovery and functional analysis, especially for transcription factors. Advanced genome sequencing technologies have enabled rapid access to entire genetic codes and their expression profiles of particular organisms, including microalgae species, which facilitate the genome-scale identification of TF-encoding genes. The list of TFs and their expression patterns, relative to non-TF genes, are useful in order to illustrate the gene regulatory networks associated with various cellular functions that can facilitate the narrowing down of candidate regulators, which may be useful for metabolic engineering in microalgal species (Figure 1). In addition to such TF-based genetic engineering strategies, recent advances in sequencing technologies have enabled us to explore the genomic diversities underpinning their cellular functions, associated with adaptation abilities to particular environments. Whole-genome sequencing of microalgal species adapting to extreme conditions has highlighted particular genetic components that may be associated with their adaptation to adverse environments, such as acidic environments [67], and low temperatures and high salinity [68], which may provide useful resources to explore genes involved in their adaptation strategies. Moreover, platforms for high-throughput phenotyping and screening may also play crucial roles in the genetic engineering of microalgal species [69]. These advances in microalgal genomics and phenomics will provide a framework for rationally designing microalgae strains with improved productivity, which could facilitate microalgae-based bioproduction.

## Data Availability

The data presented in this study are available in this article.
